# Functionalized platinum nanoparticles with surface charge trigged by pH: synthesis, characterization and stability studies

**DOI:** 10.3762/bjnano.7.175

**Published:** 2016-11-24

**Authors:** Giovanna Testa, Laura Fontana, Iole Venditti, Ilaria Fratoddi

**Affiliations:** 1 Department of Chemistry, University Sapienza of Rome, p.le Aldo Moro 5, 00185 Rome, Italy

**Keywords:** functionalized platinum nanoparticles, pH responsive materials, synthesis of metal nanoparticles, thiol functionalization

## Abstract

In this work, the synthesis and characterization of functionalized platinum nanoparticles (PtNPs) have been investigated. PtNPs were obtained by a wet redox procedure using 2-diethylaminoethanethiol hydrochloride (DEA) as capping agent. By varying the Pt/thiol molar ratio, monodispersed and stable particles with diameters in the range of 3–40 nm were isolated. The amino functionality allows neutral particles to be obtained in basic water solution and positive charged nanoparticles in neutral or acidic water solution (pH 7–2), as confirmed by DLS and ζ-potential measurements. FTIR spectroscopy, FE-SEM, DLS and ζ-potential measurements confirmed the size and showed long term water stability (up to three months) of the colloidal system.

## Introduction

Metal nanoparticles (MNPs), in particular, platinum nanoparticles (PtNPs) offer a wide range of chemico-physical properties that can be of interest for many technological applications [1–2]. For example PtNPs are of interest due to their catalytic activity [3–4], electrochemical applications [5], chemical sensing [6–9] and optical features related to surface plasmon resonance (SPR) that occurs in the ultraviolet range of electromagnetic spectrum. It has been observed that the SPR band of PtNPs generally occurs at about 220 nm and exhibits a broad peak [10]. Moreover, the presence of high atomic number atoms makes MNPs likely candidates as innovative radiosensitizers for treatment in radiotherapy [11–12]. The role of the functionalizing layer around MNPs is of primary relevance; in fact, not only solubility but also optical properties and reactivity are strongly dependent on the external layer surrounding the MNPs [13]. The functionalization of MNPs determines their interaction with the external environment and affects their colloidal stability; hydrophobic or hydrophilic thiol-based ligands have been deeply exploited [14–15]. Among others, 2-diethylaminoethanethiol hydrochloride (DEA) has been used as a stabilizing thiol for gold nanoparticles used for the immobilization of lipase [16].

Among others, hydrothermal and solvothermal techniques are well favored for the synthesis of PtNPs, together with sol–gel methods [17]. They give rise to colloidal suspensions although a fine control of the size, dispersity and the surface functionalization is quite difficult to reach. Poly(*N*-vinyl-2-pyrrolidone) (PVP) has been used as a stabilizer to protect the platinum nanoparticles [18]. However, an efficient separation of the colloid from solution could be difficult to reach [19]. Among the synthetic approaches, wet chemical synthesis offers the opportunity to introduce a selected functionalization onto the NP surface and generally give rise to monodisperse nanoparticles [20–21].

PtNPs are generally obtained from reduction of Pt(II) or Pt(IV) ions, starting from [PtCl_4_]^2−^ or [PtCl_6_]^2−^ precursors, in the presence of a strong reducing agent, to obtain the chemical reduction to Pt(0) atoms that starts the nucleation process. If a ligand, such as an organic thiol, is present in solution, it gives rise to a passivation layer that hinders the coalescence and precipitation, allowing the colloidal suspension to remain stable [22]. Among reducing agents, hydrazine and sodium borohydride are the most commonly used but also natural-origin reducing agents such as nanocrystalline cellulose from cotton or bacterial cellulose matrixes are currently being studied [23–24].

Thiol ligands have been thoroughly investigated [25–27] and particular attention has been devoted to hydrophilic ligands that confer to the PtNP stability in water suspensions and the potential applications in biotechnology, for example, in the case of mercaptosuccinic acid [28] and ammonium bearing thiols, such as trimethyl(11-mercaptoundecyl)ammonium [29]. Pt nanoparticles stabilized with 11-mercaptoundecanoic acid were developed for the detection of volatile organic compounds (VOCs) [30].

In this study, the synthesis and characterization of functionalized platinum nanoparticles has been investigated. PtNPs were obtained by a wet redox procedure, using 2-diethylaminoethanethiol hydrochloride (DEA) as a capping agent. By varying the Pt/thiol molar ratio, stable, monodisperse nanoparticles with diameters in the range of 3–40 nm were isolated. The amino functionality allows acid–base equilibria [31], and with this system, it is possible to obtain neutral particles in basic water solution and positive charged nanoparticles in acidic water solution.

## Results and Discussion

### PtNP synthesis and characterization

**Scheme 1:**
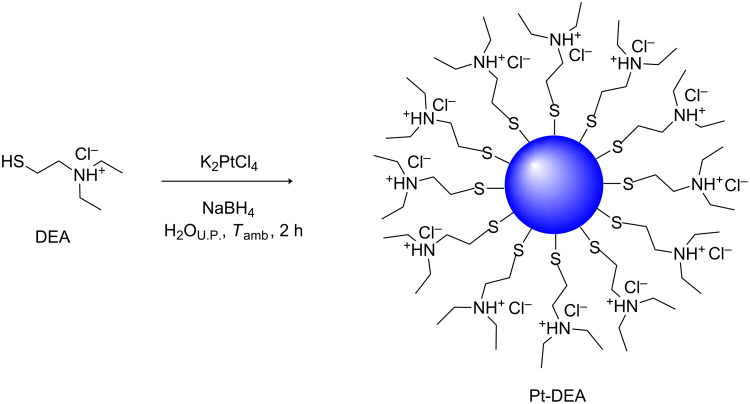
Reaction scheme for Pt-DEA nanoparticles.

The procedure used for the synthesis of PtNPs stabilized with DEA is reported herein. It was based on a wet reduction with water as a solvent [32] at room temperature. The formation process of thiol-protected Pt nanoparticles (Pt-DEA) synthesized in water solution is herein presented as a representative example, and the reaction scheme is reported in Scheme 1. Different Pt/DEA molar ratios have been used and the reduction products were analyzed in order to study the effect of this parameter on the nanoparticle diameter.

**Figure 1:**
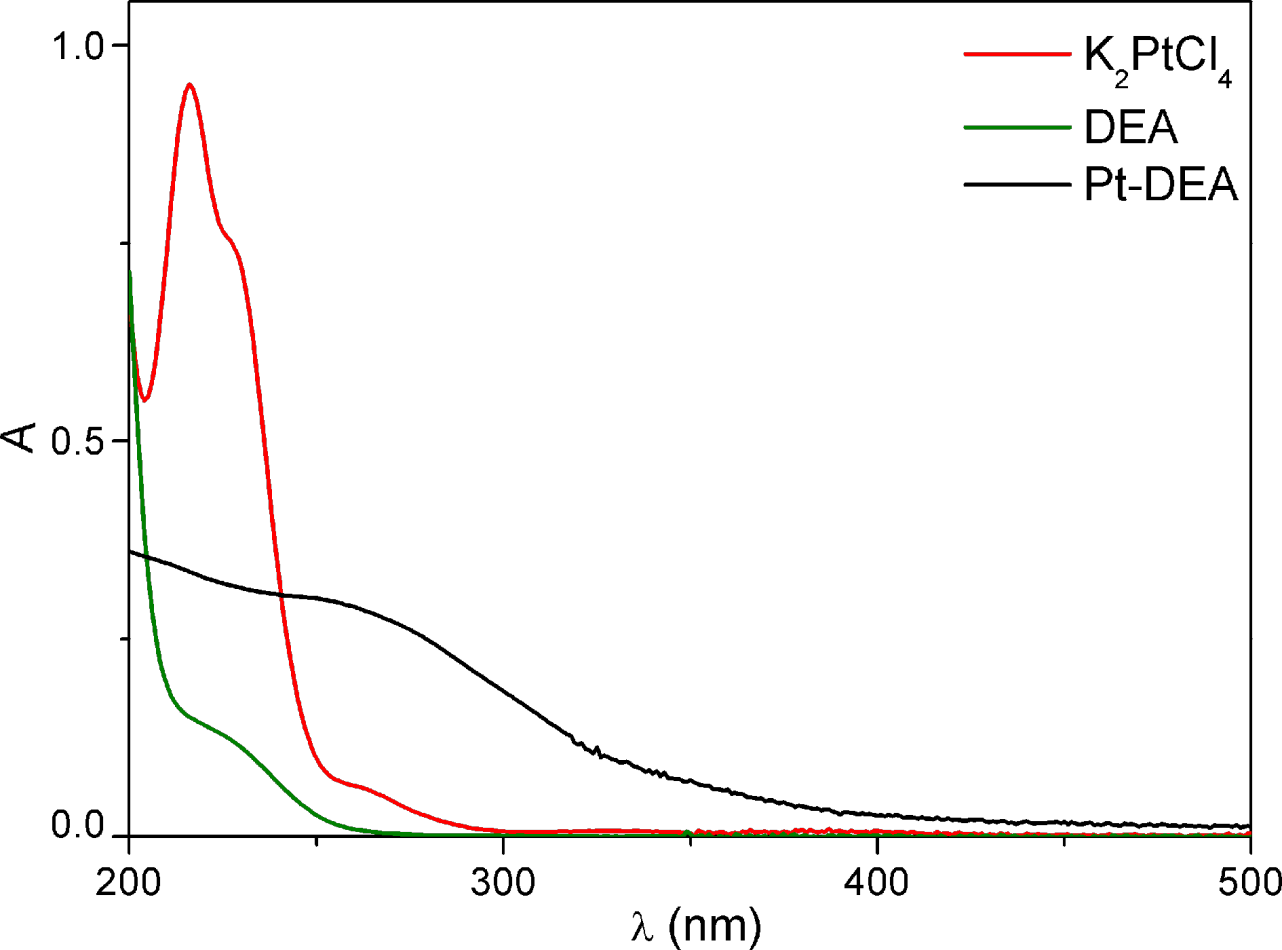
**1** UV–vis spectra in H_2_O of: K_2_PtCl_4_ (red); DEA (green); Pt-DEA (black).

The Pt(II) starting complex solution is pale yellow before mixing and shows a peak at 220 nm in the UV–vis spectrum due to the ligand-to-metal charge-transfer transition of the [PtCl_4_]^2−^ ions [33]. This is shown in Figure 1 together with the absorption features of DEA thiol.

As the NaBH_4_ reducing agent is added, the peak at 220 nm decreases and disappears within 30 min, indicating that the [PtCl_4_]^2−^ ions are completely reduced in 30 min according to Equation 1.

[1]
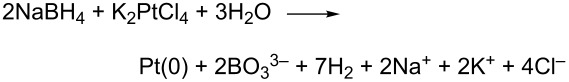


The color of the solution rapidly turns from yellow into dark brown, and the absorption of the mixture in the visible region increases, until a broad tailing peak appears at about 264 nm (see Figure 1), suggesting that the band structure of the Pt nanoparticles is formed [34].

After careful purification by centrifugation, the colloidal product was characterized by DLS, UV–vis spectroscopy, FTIR, and *ζ*-potential measurements. A FESEM study was carried out on selected samples.

**Figure 2:**
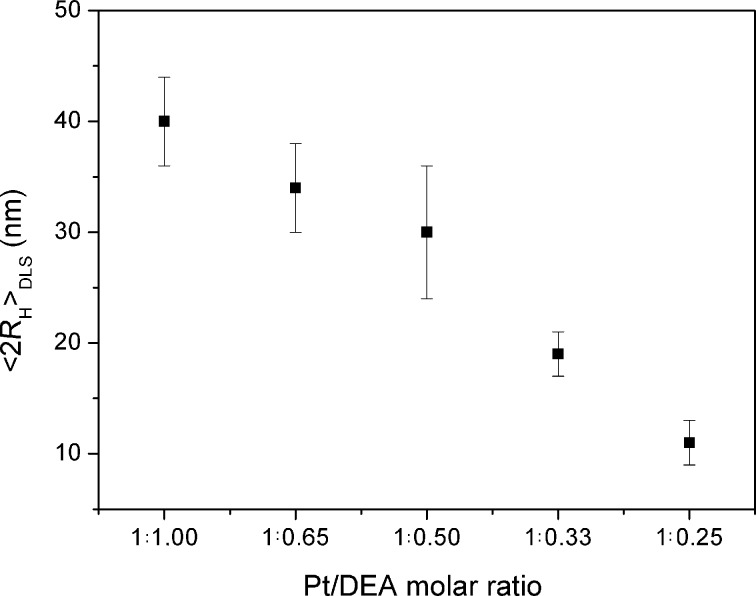
**2** Influence of Pt/DEA molar ratio: DLS results in water.

In Figure 2, the trend of the DLS hydrodynamic diameter <2*R*_H_> as a function of the Pt/DEA molar ratio used during the synthesis is reported, where a fixed Pt/NaBH_4_ molar ratio of 1:5 was maintained. It can be observed that a general decrease of the DLS-measured diameter is observed, from 40 ± 4 nm to 11 ± 2 nm ranging from 1:1 to 1:0.25 Pt/DEA molar ratio. The influence of the metal/ligand molar ratio has been thoroughly investigated in the literature for Au and Ag NPs [35] and is herein reported for the first time for Pt in the presence of the DEA ligand. Here, a complex effect can be envisaged, deriving from the presence of the thiol functionality and the ammonium end group [36] that undergoes to acidic–base equilibrium in aqueous solution.

**Figure 3:**
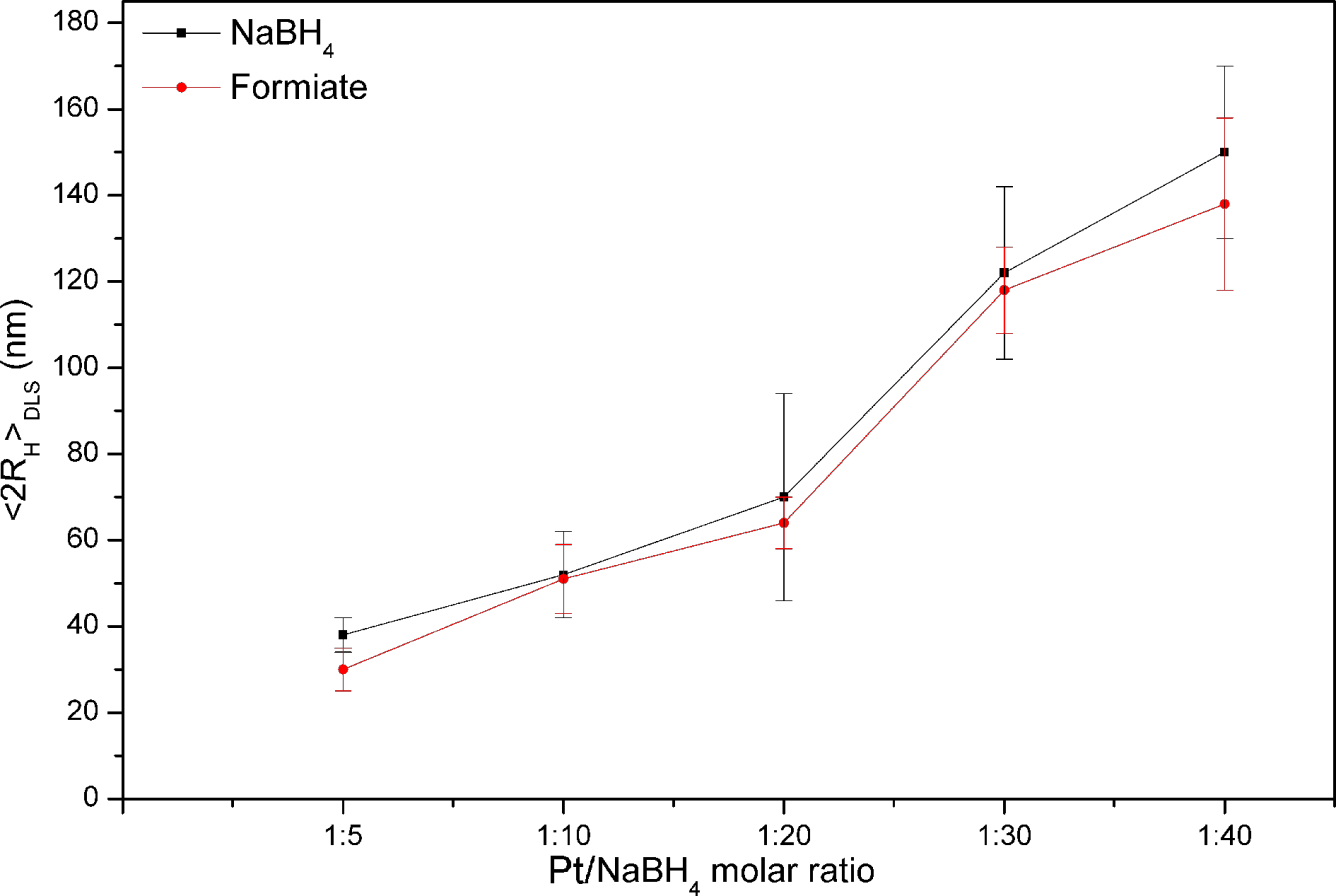
**3** Influence of Pt/reducing agent molar ration in the formation of Pt-DEA nanoparticles: DLS results in water.

The role of the reducing agent has been studied and the effect of the widely used NaBH_4_ has been compared with ammonium formiate (HCOONH_4_) [37–38]. With this aim, maintaining the Pt/DEA ratio fixed at 1:0.5, the molar ratio between metal and reducing agent has been varied as follows: Pt/NaBH_4_ 1:5, 1:10, 1:20, 1:30, 1:40 and the results were compared with the effect of HCOONH_4_. From results reported in Figure 3, it can be observed that a similar behavior has been obtained for the used reducing systems, and as a general trend, the DLS-measured diameter increases, increasing the amount of reducing agents.

The ζ-potential of Pt-DEA has been studied and values denoting a stable colloidal suspension of nanoparticles was found (±30 mV, indicating a stable colloid [39]). As observed in the literature, the role of the stable negative counter-ion is underlined from the negative values observed [40]. Measurements carried out up to three months confirmed the long-term stability of the colloidal suspension for all the samples.

**Figure 4:**
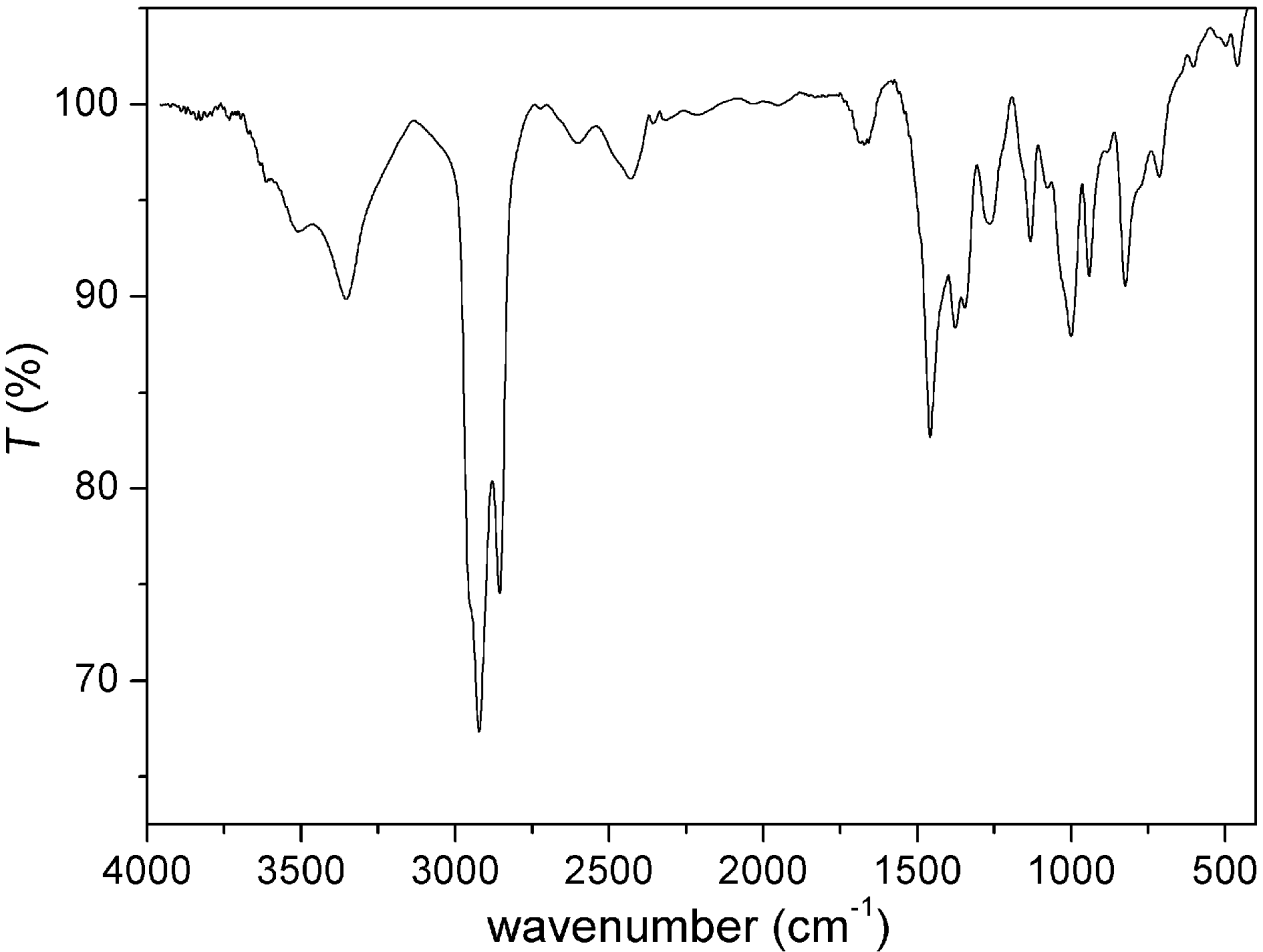
FTIR spectrum of Pt-DEA (Pt/DEA 1:1) nanoparticles (film).

**Figure 5:**
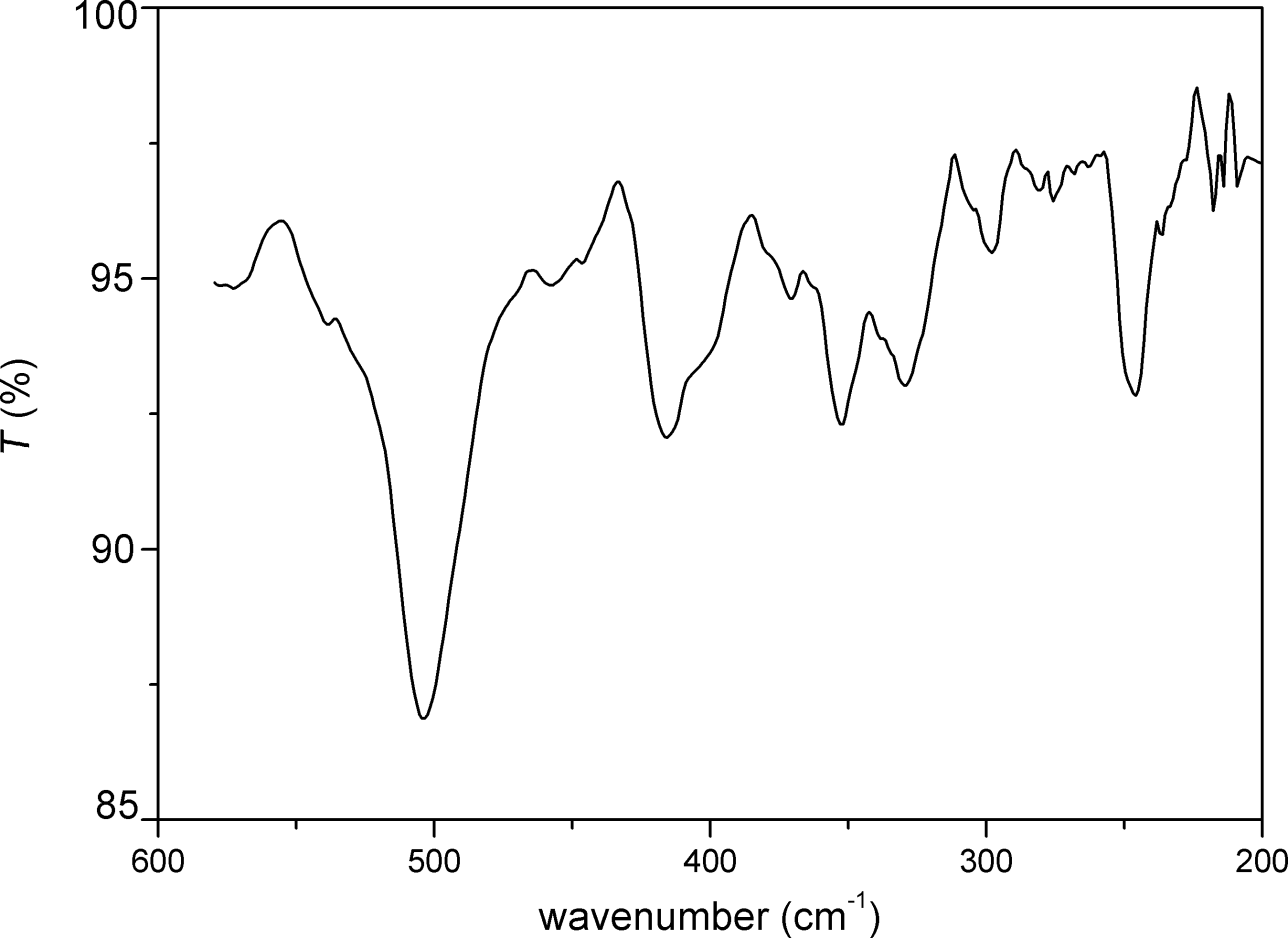
Far-IR spectrum of Pt-DEA (Pt/DEA 1:1) nanoparticles (Nujol).

The FTIR spectrum of Pt-DEA nanoparticles (Pt/DEA 1:1) is reported in Figure 4, where the intense peaks at 2927 and 2854 cm^−1^ can be attributed to the symmetric and antisymmetric stretching of –CH_2_ and –CH_3_ groups of the DEA ligand, and the peak at 3346 cm^−1^ arises from the ammonium group of DEA. The absence of the S–H stretching mode of free thiol at about 2500 cm^−1^ can be due to the absence of free unreacted thiols. In the far infrared spectrum, as reported in Figure 5, the presence of the Pt–S stretching bands at about 330 cm^−1^ confirms the functionalization on the Pt surface with DEA thiol [41–42].

**Figure 6:**
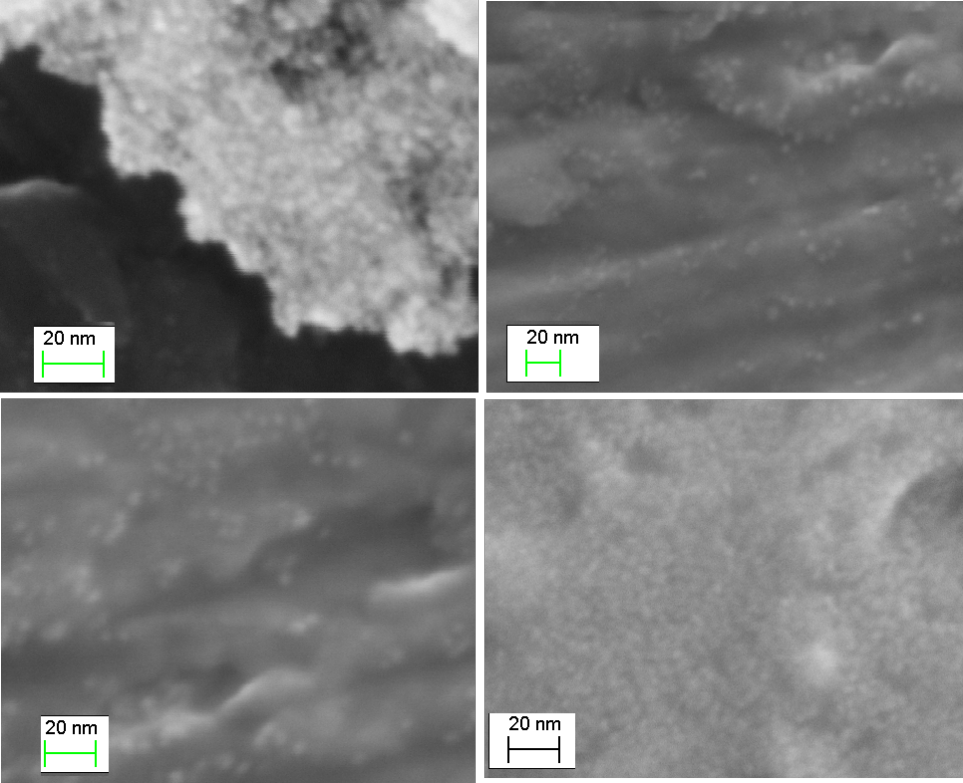
FESEM images of Pt-DEA nanoparticles (Pt/DEA 1:0.25).

FESEM images of Pt-DEA nanoparticles (reported in Figure 6 for different regions of the Pt/DEA 1:0.25 sample) evidenced the presence of small and uniform PtNPs, with diameter ≈5 nm, having a smaller diameter with respect to the DLS data, as already observed for similar compounds [43]. In fact, the hydrodynamic diameters given by DLS are usually larger than the number average diameter of the particles, and DLS overestimates the average particle sizes [44]. While FESEM is a solid state technique, in this case carried out on dried samples, DLS results take into account the dynamic equilibrium in a solution of nanoparticles [45].

### Role of pH on aggregation

**Figure 7:**
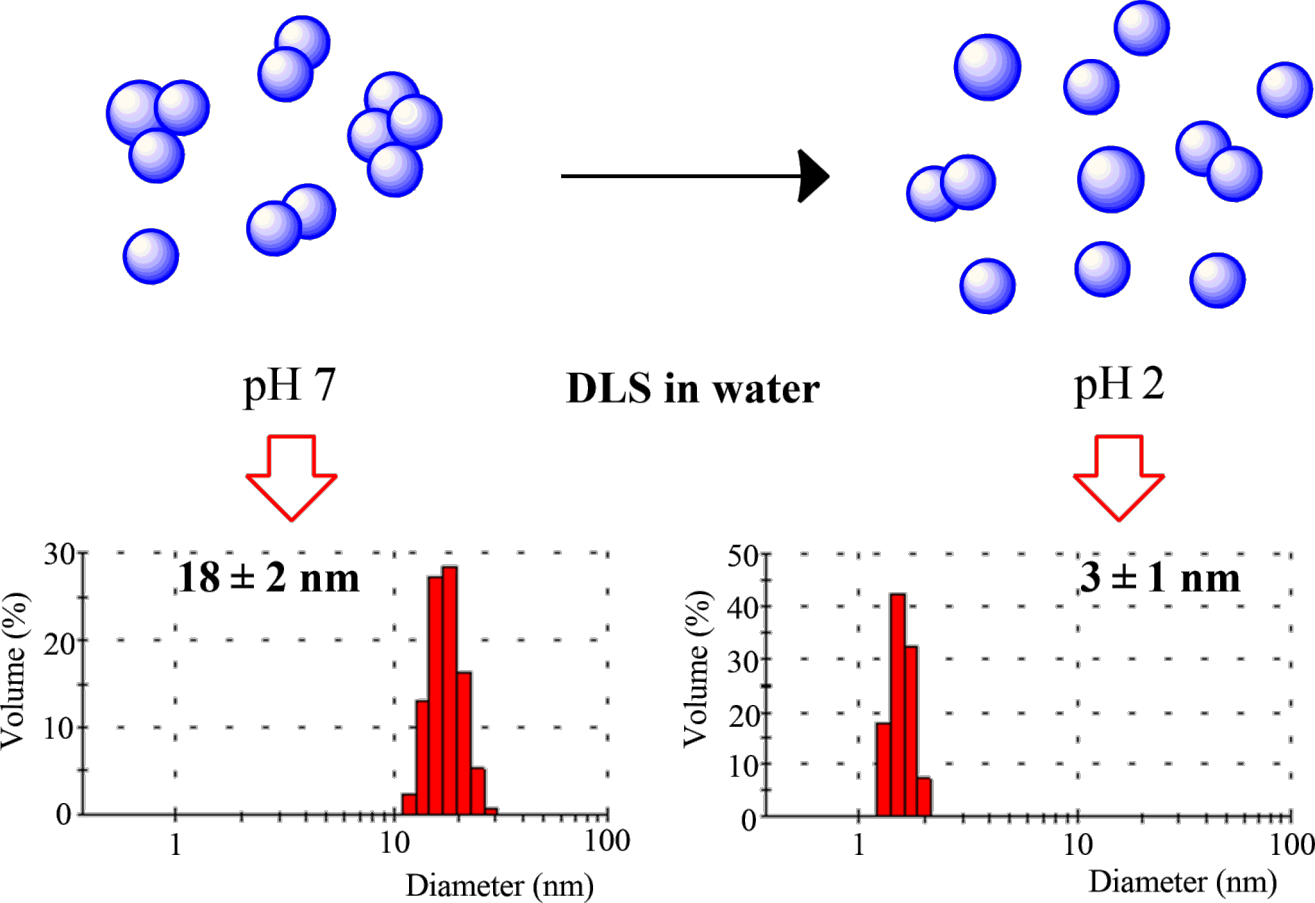
Sketch of the aggregation phenomenon together with DLS data of Pt-DEA nanoparticles (Pt/DEA 1:0.33) at different pH.

The role of pH on aggregation phenomena has been investigated for Pt-DEA nanoparticles with a Pt/DEA 1:0.33 molar ratio. In particular, at pH 7, the nanoparticles are more aggregated (18 ± 2 nm) than pH 2 (3 ± 1 nm). A chemical sketch of the aggregation phenomenon together with the DLS data are reported in Figure 7. The role of the ammonium end group could be a critical attributor to this behavior. In fact, at low pH values, the end group becomes positively charged and tends to disaggregate vicinal nanoparticles, whereas at higher pH values, the amine group becomes predominant in the acidic–base equilibrium and aggregation occurs.

## Conclusion

In this work stable, monodisperse PtNPs with small diameter have been obtained. Among the various Pt/DEA molar ratios, the 1:0.25 sample was studied in detail. The FTIR and far IR measurements confirmed the formation of the Pt–S bond on the PtNP surface. The DLS characterization demonstrated the long-term stability of the colloidal system up to three months and the possibility to tune the aggregation phenomenon of the PtNPs by varying the pH due to modulation of the metal surface charge.

## Experimental

### Instrumentation

UV–vis spectroscopy was performed in H_2_O solutions using quartz cells with a Varian Cary 100 Scan UV–vis spectrophotometer. The diameter and the diameter distribution of MNPs in H_2_O solution were investigated at 25 ± 0.2 °C (pH 7 and pH 2) by means of dynamic light scattering (DLS) technique by using a Malvern Nano ZS90 scattering apparatus (Malvern Instruments Ltd., Worcestershire, UK). The correlation functions were collected at 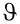 = 90° relative to the incident beam, and delay times from 0.8 µs to 10 s were explored [46–47]. Non-negative least-squares (NNLSs) [48] or CONTIN [49] algorithms, supplied with the instrument software, were used to fit correlation data. The ζ-potential was calculated from the measured electrophoretic mobility by means of the Smolukovsky equation [50]. FTIR and far IR spectra were recorded on cast-deposited films from Nujol using KRS-5 cells with a Bruker Vertex 70 spectrophotometer. Field emission scanning electron microscopy (FE-SEM) images and energy dispersion spectroscopy (EDS) were acquired with the Auriga Zeiss instrument (resolution 1 nm, applied voltage 6–12 kV) on freshly prepared films drop-cast from H_2_O solution on a metallic sample holder. A MiniSpin (Eppendorf) centrifuge was used for purification of PtNP samples (13000 rpm, 20 min, 15 times with deionized water). Deionized water was obtained from Zeener Power I Scholar-UV (18.2 MΩ).

### Materials

Potassium tetrachloroplatinate (K_2_PtCl_4_ 98.5%, Sigma-Aldrich), 2-diethylaminoethanethiol hydrochloride (HS(CH_2_)_2_N(CH_2_CH_3_)_2_·HCl, 98% Sigma-Aldrich, DEA), sodium borohydride (NaBH_4_, 99% Sigma-Aldrich) were used as received.

PtNPs were prepared at room temperature in a single-phase system by modifying previous literature reports [51]. In particular, PtNPs-DEA were prepared with Pt/DEA molar ratios ranging from 1:1 to 1:0.25. In a typical procedure, 0.200 g of K_2_PtCl_4_ (4.8 × 10^−4^ mol) were dissolved in 10 mL of deionized water at pH 2 and mixed with a solution of DEA in deionized water (0.0815 g, 4.8 × 10^−4^ mol in 10 mL for the 1:1 Pt/DEA molar ratio). After stirring the mixture under argon for 10 min at room temperature, a NaBH_4_ solution in deionized water (0.177 g, 4.8 × 10^−3^ mol in 10 mL) was added dropwise. The reaction mixture was allowed to react for 20 h and the obtained suspension was centrifuged with deionized water ten times (10 min, 15000 rpm), recovering the black solid from the supernatant (48 wt % yield ).

The following are the experimental data for the various PtNPs-DEA samples:

- PtNPs-DEA 1 (Pt/DEA 1:1): DLS (2*R*_H_ [nm], H_2_O): 40 ± 4; IR (ν [cm^−1^], Nujol): 3346, 1006, 948 (Et_3_N^*^–H), 325, 350 (Pt–S); ζ-potential ([mV], H_2_O): −27.
- PtNPs-DEA 2 (Pt/DEA 1:0.65): DLS (2*R*_H_ [nm], H_2_O): 34 ± 4; IR (ν [cm^−1^], Nujol): 3345, 1008, 950, 323, 350 (Pt–S); ζ-potential ([mV], H_2_O): −25.
- PtNPs-DEA 3 (Pt/DEA 1:0.5): DLS (2*R*_H_ [nm], H_2_O): 30 ± 6; IR (ν [cm^−1^], Nujol): 3342, 1006, 950; ζ-potential ([mV], H_2_O): −27.
- PtNPs-DEA 4 (Pt/DEA 1:0.33): DLS (2*R*_H_ [nm], H_2_O): 18 ± 2; IR (ν [cm^−1^], Nujol): 3344, 1008, 948; ζ-potential ([mV], H_2_O): −25.
- PtNPs-DEA 5 (Pt/DEA 1:0.25): DLS (2*R*_H_ [nm], H_2_O): 11 ± 2; IR (ν [cm^−1^], Nujol): 3342, 1007, 948; ζ-potential ([mV], H_2_O): −27.
